# Exploring the temporal dynamics of rumen bacterial and fungal communities in yaks (*Bos grunniens*) from 5 days after birth to adulthood by full-length 16S and 18S rRNA sequencing

**DOI:** 10.3389/fvets.2023.1166015

**Published:** 2023-06-21

**Authors:** Shuli Yang, Guangrong Zhang, Zaimei Yuan, Shichun He, Rongjiao Wang, Jieyi Zheng, Huaming Mao, Jianmin Chai, Dongwang Wu

**Affiliations:** ^1^Guangdong Provincial Key Laboratory of Animal Molecular Design and Precise Breeding, College of Life Science and Engineering, Foshan University, Foshan, China; ^2^Key Laboratory of Animal Nutrition and Feed Science of Yunnan Province, Yunnan Agricultural University, Kunming, China; ^3^Kunming Animal Disease Prevention And Control Center, Kunming, China; ^4^Panzhihua Academy of Agricultural and Forestry Sciences, Panzhihua, China

**Keywords:** rumen microbiology, yak, microbiome, bacteria, fungi

## Abstract

The rumen of ruminants is inhabited by complex and diverse microorganisms. Young animals are exposed to a variety of microorganisms from their mother and the environment, and a few colonize and survive in their digestive tracts, forming specific microflora as the young animals grow and develop. In this study, we conducted full-length sequencing of bacterial and fungal communities in the rumen of pastured yaks of different ages (from 5  days after birth to adulthood) using amplified sequencing technology. The results showed that the rumen microflora of Zhongdian yaks changed gradually from 5 to 180  days after birth and tended to stabilize at 2  years of age. The rumen of adult yaks was the most suitable for the growth and reproduction of most bacteria. Bactria diversity of the yak rumen increased gradually from 5  days after birth to adulthood. With the growth of yaks, different dominated bacteria were enriched in different groups, but *Prevotella* remained highly abundant in all groups. The yak rumen at 90  days of age was the most suitable for the growth and reproduction of most fungi, and 90 days of age could be a cut-off point for the distribution of fungal communities. Fungal *Thelebolus* was the firstly reported in yak rumen and was enriched in the yak rumen of 90  days after birth. The most abundant and balanced fungal genera were found in adult yaks, and most of them were only detected in adult yaks. Our study reported on the rumen bacterial and fungal communities of Zhongdian yaks grazed at different ages and provided insights into the dynamic changes of dominant microflora with yak growth.

## Introduction

Gut microbiomes are inseparable from their animal host (1, 2), affecting the health and proper functioning of their host. Ruminant rumen microbes degrade complex plant polysaccharides into compounds which can be absorbed and utilized by ruminants (3). This critical process not only converts plant fiber in forage grass into milk and meat for human consumption but also promotes the reproduction and growth of rumen microorganisms. In young ruminants, microflora plays an important role in the development of the rumen wall and in nutrient absorption processes. Zhongdian yak (*Bos grunniens*) is a characteristic breed of cattle of Chinese Yunnan Province, playing a vital role in the economic, genetic, and ecological diversity of the Qinghai Tibet Plateau area (4, 5). They live in a low-temperature hypoxic high-altitude environment and have a higher quality production of milk than that of dairy cows (6). Investigation of yak rumen microbes is important to understand how rumen microbiome are associated with their specific phenotypes.

It has been well-proven that the rumen microbiome is related to altitude adaptation and milk performance traits. The rumen microbiome diversity of Zhongdian yak is significantly higher than that of cattle and buffalo, who live at an altitude of 1,100 m (7). Interactions of Proteobacteria-choline and Firmicutes-myristic acid in the rumen cause to changes in milk fat percentage in yaks (6). Furthermore, the rumen microflora of ruminants undergoes gradual changes from birth, primarily marked by an increase in anaerobic microorganisms and a decrease in aerobic and facultative anaerobic microorganisms (8). The rumen microflora of calves undergoes changes with growth and dietary structure, while the rumen physiological environment facilitates the interaction between microorganisms (9). Facultative anaerobic flora typically exhibit higher levels during the first day of a calf’s life and then decrease to a stable abundance after 6–8 weeks (10). Anaerobic fungi appear in the rumen of ruminants shortly after birth, and communities of bacteria, fungi, and archaea form in the rumen of animals at 7 days of age (11, 12). The microflora of young ruminants was similar, but the abundance and diversity of the microflora also changed with the change in dietary structure and the increase in feed intake (13). Bacterial and fungal changes in the calve rumen are also affected by the natural grazing patterns of calves. Although researchers have explored the effects of the rumen microbiome on plateau adaptation and milk-production performance, an understanding of the microbial colonization process in the life of grazing yak calves remains elusive.

Exploring the temporal dynamics in microbial communities in yaks from birth to adulthood can aid to establish microbial interactions during rumen development. Hence, in this study, the full length of bacterial 16S rDNA and fungal ITS2 of rumen fluids from grazing Zhongdian yaks at 5, 45, 90, 180, and 720 days of age were sequenced to investigate the temporal dynamics of rumen microbiome. Our results described rumen bacterial and fungal compositions and their temporal dynamics (newborn to adulthood) in Zhongdian yaks, which allow us to understand the importance of rumen microbiome in yaks. Rumen fluid was collected from 3 yaks at 5, 45, 90, and 180 days after birth. Rumen fluid was collected from 6 yaks 720 days after birth. Bacterial 16S rDNA and fungal ITS2 sequencing were performed to investigate the changes in rumen microbial composition, diversity, core microorganisms, and anti-inflammatory characters in different ages of Zhongdian yak. Our results described rumen bacterial and fungi compositions and the dynamics occurring with ages (newborn to adulthood) in Zhongdian yaks.

## Materials and methods

### Experimental animals

The animal trial was conducted in the natural pasture of Tiancheng Lun Zhu Agricultural Products Development Co., Ltd. in the north of Shangri-La County, which is a double-row fully open barn with free grazing open feeding mode for the experimental yaks. There are drinking water supply points in the pasture, which can ensure the yaks’ drinking water. In July 2019, 10 female yaks were selected from the Zhongdian yak herd which were simultaneously estrous and inseminated in 2018 (average gestation period of 271 days). After the calves were born, six yak calves in similar conditions were selected for sampling. The calves were separated from their dams on day 5 after birth. Rumen fluids were collected from each yak at 5, 45, 90, and 180 days after the birth. The experimental yaks were divided into five groups according to their age: D5 (5-day after birth), D45 (45 days after birth), D90 (90 days after birth), D180 (180 days after birth), and D720 (adult yaks; Supplementary Table S1). The nutrient compositions of the fed diets are shown in Supplementary Table S2.

An oral stomach tube was used for collection of rumen fluid samples. In brief, 2 h after morning grazing, a tube was inserted into the rumen, and a vacuum sampler was used to pump rumen fluid. For each animal, 30 ml of rumen fluid was collected, and divided into three parts which were placed in 10 ml polypropylene tubes. Then, all samples were rapidly stored in liquid nitrogen, and brought back to the laboratory for storage in a refrigerator at −80°C. Due to some calves dead during the experiment, the number of yaks was reduced to three. Six adult yaks were randomly collected from naturally grazing yaks that were birthed in 2017, and the rumen fluid was collected using the same method.

### DNA extraction and sequencing

The EZNA Stool DNA Kit (Omega Bio-Tek, Norcross, GA, USA) was used to extract microbial community DNA according to the manufacturer’s instructions. DNA was quantified using the Qubit dsDNA BR Assay kit (Invitrogen, USA) and quality was assessed by running an aliquot on a 1% agarose gel. To amplify variable regions V1-V9 of the bacterial 16S rRNA gene, degenerate PCR primers 27F (5′-AGRGTTYGATYMTGGCTCAG-3′) and 1492R (5′-RGYTACCTTGTTACGACTT-3′) were used (14). Additionally, degenerate PCR primers ITS3 (5′-GCATCGATGAAGAACGCAGC-3′) and ITS4 (5′-TCCTCCGCTTATTGATATGC-3′) were used to amplify the ITS2 of the internal transcribed spacer (ITS) region (15). Both forward and reverse primers were tagged with Illumina adapter, pad, and linker sequences. PCR enrichment was carried out in a 50 μl reaction containing 30 ng template, fusion PCR primer, and linker sequences, using PCR master mix. The PCR cycling conditions were as follows: 94°C for 3 min, 30 cycles of 94°C for 30 s, 56°C for 45 s, and 72°C for 45 s, and a final extension for 10 min at 72°C. PCR products were purified using AmpureXP beads and eluted in elution buffer. The Agilent 2100 Bioanalyzer (Agilent, USA) was used to qualify the libraries. Validated libraries were sequenced using the Illumina MiSeq platform (BGI, Shenzhen, China) following Illumina’s standard pipelines. Bacterial and fungal sequences from this project have been deposited in the National Center for Biotechnology Information (NCBI) Short Read Archive (SRA) under the BioProject number PRJNA630991.

### Bioinformatics and data analyses

The raw paired-end reads from the sequencer were merged and filtered to eliminate adapter pollution and low-quality readings, obtaining clean reads. Further processing and quality controls were performed using mothur v1.42.1 according to the most recent versions of our lab’s standard analysis pipelines, adapted from the Schloss lab protocol (16). Chimeric sequences were identified and removed using the UCHIME algorithm in mothur. Subsequently, tags were clustered into operational taxonomic units (OTUs) with a 97% sequence similarity. Taxonomic ranks were assigned to the representative sequences of the OTUs using the Ribosomal Database Project (RDP) Naive Bayesian Classifier v2.2. Alpha diversity, beta diversity, and the screening of different species were then analyzed based on the OTUs and taxonomic ranks.

Tags were clustered into operational taxonomic units (OTUs) using USEARCH (v7.0.1090) software. The taxonomic classification of OTU representative sequences was performed using Ribosomal Database Project (RDP) Classifier v.2.2, trained on the Greengene_2013_5_99 database. A 0.5% confidence value was used as the cutoff. Filtered tags were then clustered into OTUs at 97% similarity, where the OTU number per sample primarily represented the degree of sample diversity. The OTUs of each group were listed, and Venn diagrams were generated using Venn Diagram Software R (v3.1.1). Common and specific OTU IDs were summarized. Based on the abundance of OTUs, the relative abundance of each OTU in each sample was calculated. Principal component analysis (PCA) of the OTUs was performed using the relative abundance values. Good’s coverage, alpha diversities (including Inverse Simpson and Shannon indices), richness (observed number of OTUs), and evenness (Shannon evenness) were calculated using Mothur V.1.31.2. Beta diversity analysis was also performed. Since there were differences in sequencing depth among samples, normalization was introduced by randomly extracting sequences according to the minimum sequence number for all samples. The extracted sequences were used to generate a new “OTU table biom” file, and the beta diversity distance was calculated based on this file. OTUs were annotated based on the Kyoto Encyclopedia of Genes and Genomes (KEGG) version 2018.01. The outputs of diversity analysis were visualized using the “ggplot2” package in R (v3.6.0), which is available online at https://www.r-project.org/.

The Linear Discriminant Analysis (LDA) Effect Size (LEfSe 1.1.01), an analytical tool used to discover and interpret biomarkers in high-dimensional data, was employed to identify the signature bacteria associated with growth stages and intestinal segments. A criterion of LDA score > 2 was used to determine significant effect size. The signature bacteria were visualized in a heatmap using the “pheatmap” function in R.

For assessing microbial interactions within treatments, network analysis was conducted by calculating all possible Pearson rank correlation coefficients (ρ) between microbial pairs. To minimize spurious associations, we considered a valid co-occurrence between two different taxa if the correlation coefficient was above 0.6 or below −0.6 and statistically significant. The subnetworks within the regimes were generated based on betweenness clustering calculated using the Girvan-Newman algorithm.

## Results

### Sequencing of the yak rumen at different ages

**Figure 1:**
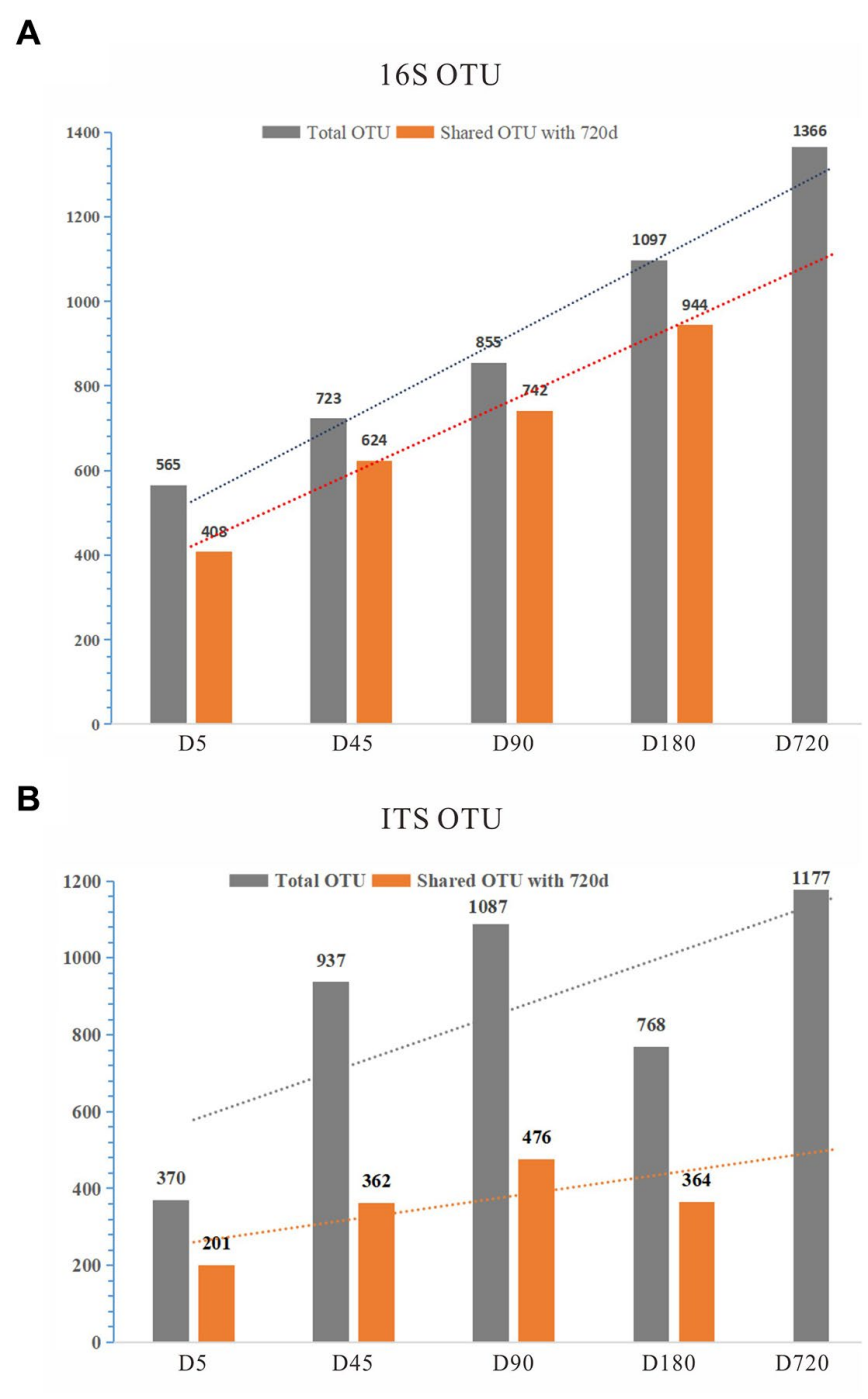
The statistics of OTUs of 16S and ITS. **(A)** OTUs of 16S; **(B)** OTUs of ITS.

To explore the rumen microbial community of grazing yaks at different growth stages, we sequenced the full length of 16S rDNA and ITS for each sample using the Illumina HiSeq 2500 sequencing platform. A total of 164,397 16S rDNA sequences were detected from all samples, with an average of 9,133 sequences per sample, and were clustered into 1,709 OTUs with 97% similarity. With the increase of age, the number of OTUs detected in the rumen increased linearly. By comparing with the D720, the number of overlapped OTUs was 408 (72.21%) at the D5, 624 (86.31%) at the D45, 742 (86.78%) at the D90, and 944 (86.05%) at the D180, indicating that a large number of microorganisms existed stably in the rumen from 45 days until adulthood (Figure 1A; Supplementary Table S3).

For the ITS analysis, 1,009,208 ITS sequences were detected from all samples, with an average of 56,067 ITS sequences per sample, and clustered to 2,525 fungal OTUs. By comparing with the D720, the number of overlapped OTUs was calculated, which was low in each group, indicating that the fungi of the yak rumen were variable during their growth (Figure 1B; Supplementary Table S3). The detected OTUs were labeled to construct changes in the rumen microflora of Zhongdian yaks at different growth stages.

### Bacterial community diversity and the temporal dynamics of the rumen signature bacteria in yak

**Figure 2:**
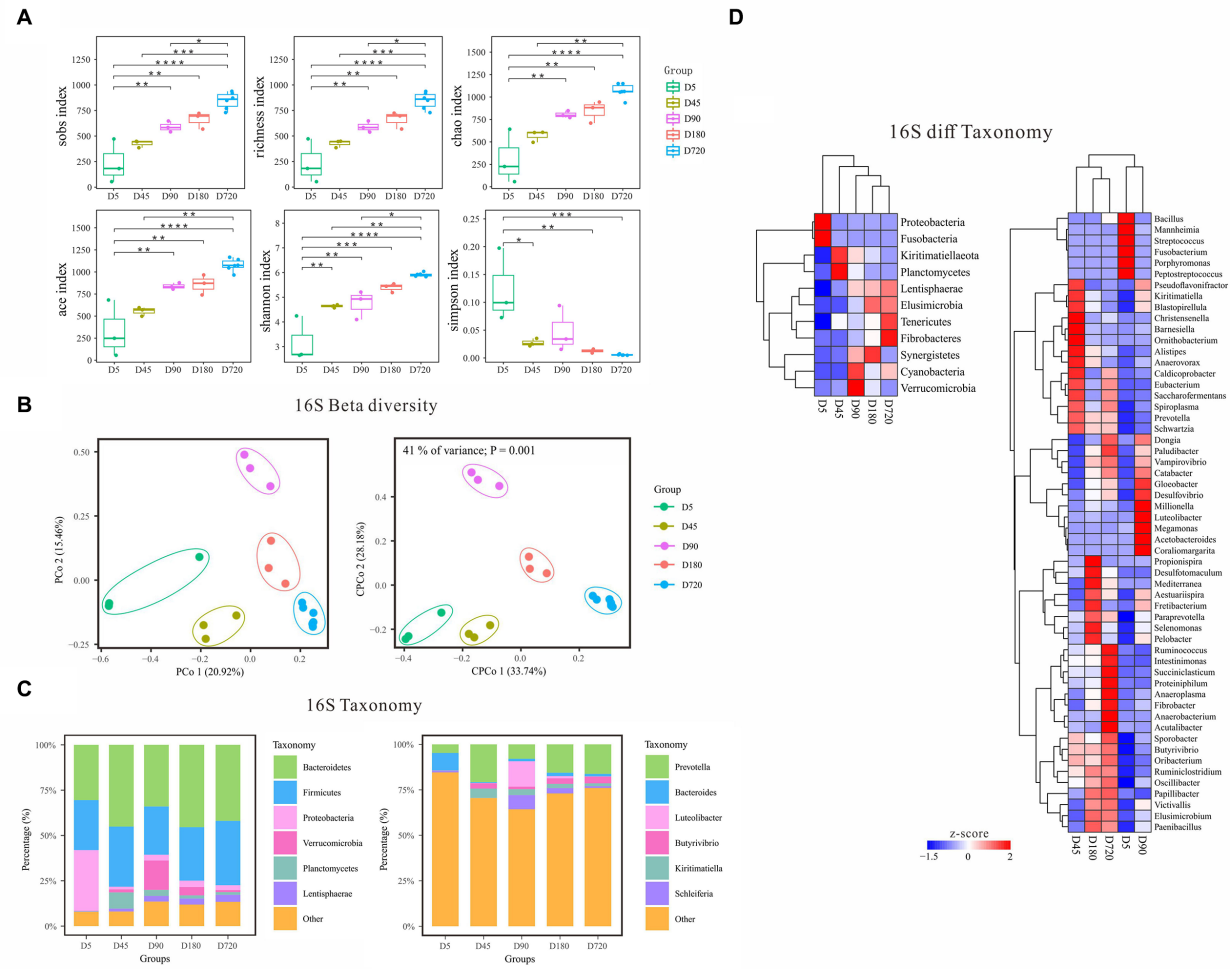
Characteristics of rumen bacterial communities of Zhongdian yaks at different ages. **(A)** Alpha diversity of bacterial communities; **(B)** Beta diversity of bacterial communities; **(C)** Composition of bacterial communities; **(D)** Phylum and genus of bacteria with significantly different abundance in different groups (ns *p* > 0.05, *p = 0.01–0.05,**p < 0.01,***p < 0.001,****p < 0.0001).

According to the alpha diversity analysis of 16S rDNA data, the biodiversity of bacteria in the yak rumen gradually increased from the D5 and reached the richest in the adult (Figure 2A). The Sobs, Richness, Chao, Ace, and Shannon indices were the highest in the adult (D720) and the lowest at D5 which gradually increased with the growth of the yak. In contrast, the Simpson index was the lowest in the adult group and the highest at D5, which gradually decreased with the growth of the yak, indicating that the rumen fluid of adult yaks was more suitable for the growth and reproduction of bacteria. Beta diversity analysis of PCoA and cPCoA showed significant differences in community distribution among different ages, indicating that there were notable differences in rumen bacterial community compositions in different yak growth stages (Figure 2B).

The dominant rumen phyla (relative abundance of all groups >10) were Bacteroidetes and Firmicutes across all ages, followed by Proteobacteria, Verrucomicrobia, Planctomycetes, Kiritimatiellaeota, Lentisphaerae, and Cyanobacteria (Figure 2C). Notably, Proteobacteria was enriched at D5, while Verrucomicrobia was higher at D90. At the genus level, *Prevotella* was the absolute dominant genus (relative abundance of all groups >50). Other genera with higher abundance at a specific age were observed. For example, *Bacteroides* was high at D5, while *Luteolibacter* was enriched at D90.

Linear discriminant analysis Effect Size (LEfSe) analysis was performed to identify the signature bacteria differentiating ages at both the phylum and genus level. At the phylum level, a total of 11 significantly different abundance phyla were detected among different ages (Figure 2D). Proteobacteria and Fusobacteria(6.69%) were higher at D5 and kept lower abundances at other ages. Planctomycetes and Kiritimatiellaeota had the highest abundance at D45 and gradually decreased from D45 to D720. Verrucomicrobia and Cyanobacteria were highly abundant at D90. The relative abundance of Synergistetes reached the peak at D180. The relative abundance of Lentisphaerae, Elusimicrobia, Fibrobacteres, and Tenericutes gradually increased from D5 to adulthood. At the genus level, *Prevotella* and *Butyrivibrio* were highly abundant in D45(15.94 and 4%), D90(19.06 and 2.7%), and D720(15.4 and 3.03%), and *Luteolibacter* (14.20%) was only highly abundant at the D90. The abundance of *Kiritimatiella* was the highest in the D45 and declined from D45 to D720, and *Streptococcus* was only highly abundant at the D5. Some signature bacterial genera were highly enriched in a specific age, including *Porphyromonas*, *Fusobacterium*, *Mannheimia*, and *Peptostreptococcus* at the D5, and *Christensenella*, *Barnesiella*, and *Ornithobacterium* at the D45, and *Megamonas*, *Millionella*, and Coraliomargarita at the D90, and *Propionispira* at the D160. In addition, the abundance of *Victivallis*, *Paraprevotella*, *Fibrobacter*, *Vampirovibrio*, and *Paludibacter* also increased gradually with the growth of the yak.

### Fungal community diversity and the temporal dynamics of fungi in the rumen of the yak

**Figure 3:**
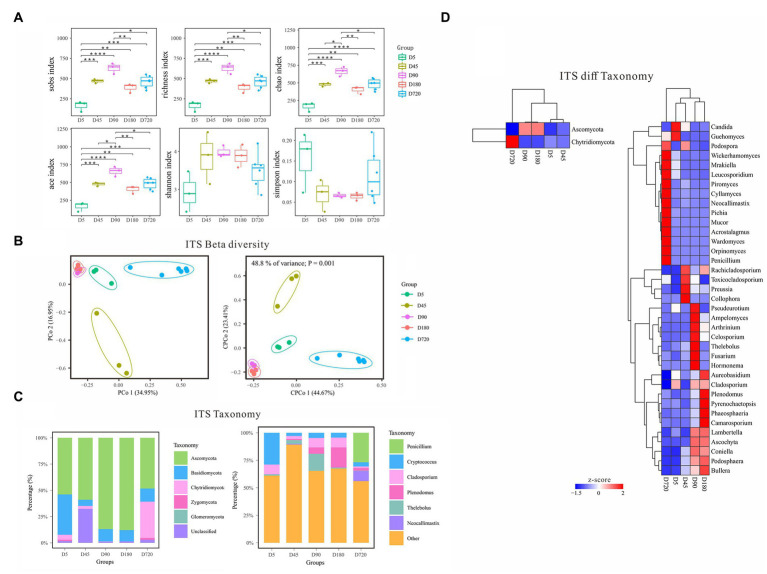
Characteristics of rumen fungal communities of Zhongdian yaks at different ages. **(A)** Alpha diversity of fungal communities; **(B)** Beta diversity of fungal communities; **(C)** Composition of fungal communities; **(D)** Phylum and genus of fungi with significantly different abundance in different groups (ns *p* > 0.05, *p = 0.01–0.05,**p < 0.01,***p < 0.001,**** *p* < 0.0001).

With the increasing age of the yak, the diversity of fungi gradually increased from D5 to the D90, and then decreased until D180, and slightly increased in adult yaks (Figure 3A). According to the *alpha* diversity analysis of ITS data, the Sobs, Richness, Chao, and Ace indices were the highest on D90 and the lowest at the D5, which gradually increased from D5 to D90, then decreased from D90 to D180, and finally increased in the D720 group. The Shannon index was also the lowest at the D5 but quite high in both D45 and D180, and the Simpson index was low in both D45 and D180 but the highest at the D5. The *alpha* diversity results showed that the rumen fluid of the D90 was the most suitable period for the growth and reproduction of most fungi and had the richest fungi biodiversity. In contrast, the rumen fluid of the D5 was the least suitable period for fungi. *Beta* diversity analysis of PCoA and cPCoA showed that rumen fluid samples of yak at different ages were separated except the D90 group and D180 were extremely similar, indicating that the changing of fungal community experienced variousness periods across the whole development of yak (Figure 3B).

The predominant fungal phyla included Ascomycota across all ages, followed by Basidiomycota, and Chytridiomycota (Figure 3C). At the same time, the dominant fungal phyla were influenced by ages, such as higher abundance of Basidiomycota at D5 and greater abundance of Chytridiomycota at D720. The dominant fungal genera mainly included *Cryptococcus Cladosporium*, *Plenodomus*, *Penicillium*, *Thelebolus*, *Saccharicola*, *Preussia*, and *Neocallimastix*, which were associated with ages. For example, *Cryptococcus* was abundance value at D45(29.3%), *Plenodomus* was over-represented at D180(18.36%), and *Penicillium* was abundance value at D5(26.7%; Figure 3C).

LEfSe analysis was also performed to detect the fungal phyla and genera differentiating ages (Figure 3D). At the phylum level, Ascomycota had the highest abundance from D45(53.29%) to D180(87.86%), and Chytridiomycota was only highly abundant at D5(34.49%). At the genus level, *Cladosporium* had a high abundance at D45(8.89%), D180(8.62%), and D720(8.93%). *Plenodomus* had the highest abundance at D720(18.36%) and followed at D90(5.7%), and *Penicillium* only had a high abundance at D5(26.7%). The abundance of *Thelebolus* was the highest at D180(15.47%) and gradually increased from D5(0.27%) to D90(3.4%), and that of *Preussia* was the lowest at D45(0.16%). *Neocallimastix* only had a high abundance at D5(9.31%). Other non-dominant fungal genera were only highly enriched in one specific age, including *Collophora* and *Rachicladosporium* at D90, and *Ascochyta*, *Fusarium*, and *Hormonema* at D90, and *Pyrenochaetopsis*, *Ascochyta*, *Phaeosphaeria* at D180, and *Orpinomyces*, *Piromyces*, *Cyllamyces*, *Mrakiella*, and *Acrostalagmus* at D720.

### Network analysis for bacterial and fungal communities in the yak rumen

**Figure 4:**
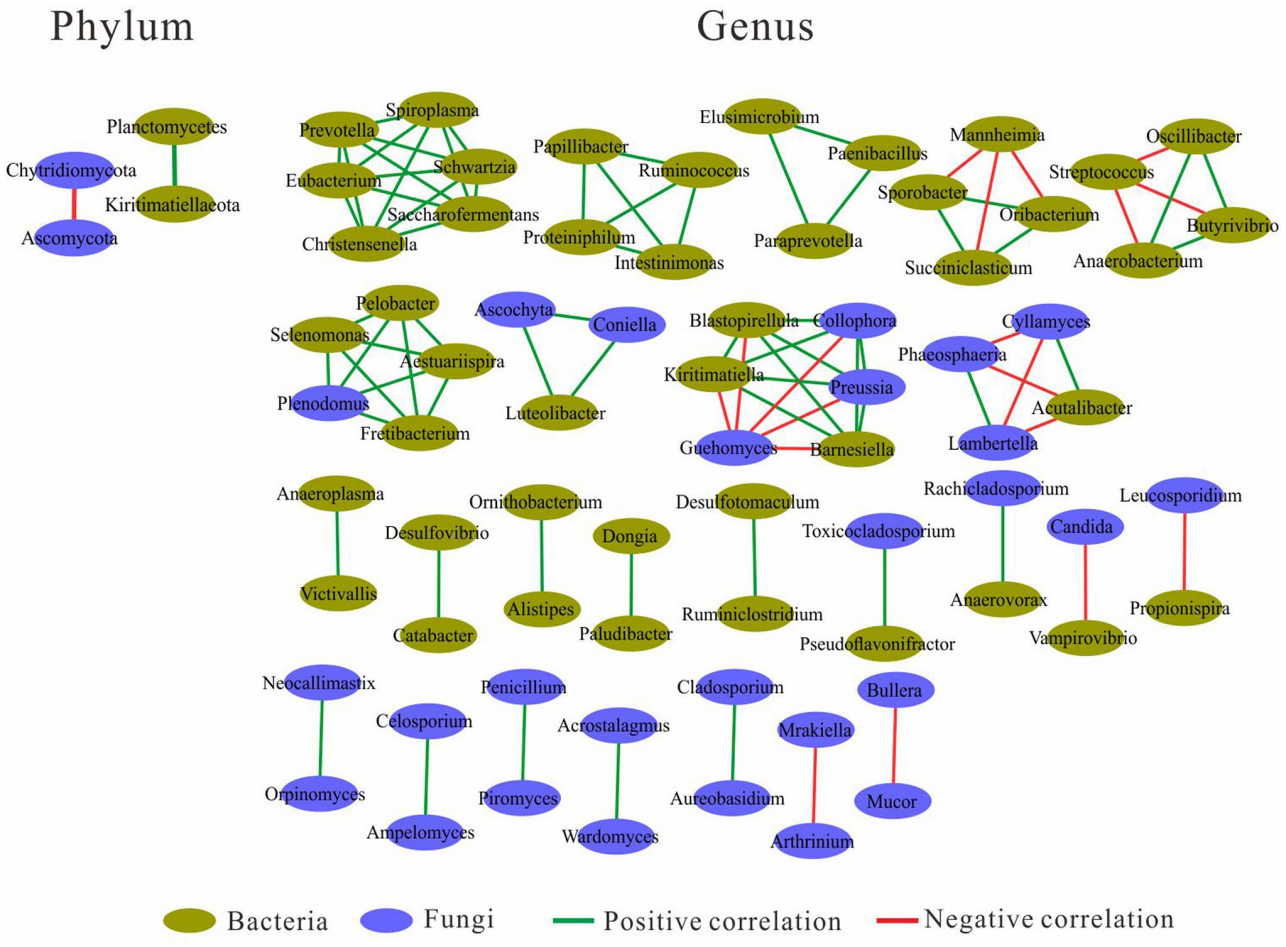
Correlation between bacterial and fungal communities.

Network analysis was performed at both the phylum and genus level (Figure 4). At the phylum level, the fungal Ascomycota was negatively correlated with fungal Chytridiomycota, and the bacterial Planctomycetes was positively correlated with bacterial Kiritimatiellaeota. At the genus level, there were four genus clusters consisting of bacteria and fungi, the clusters dominated by “*Selenomonas* and *Plenodomus*” and “*Luteolibacter* and *Ascochyta*” were all positively correlated with other genera in the same cluster, and the clusters dominated by “*Kiritimatiella* and *Preussia*” and “*Acutalibacter* and *Cyllamyces*” were negatively correlated with other genera in the same cluster. In the cluster dominated by “*Acutalibacter* and *Cyllamyces*,” the negative correlations were caused by both *Cyllamyces* and *Acutalibacter*. There were five clusters consisting of 21 bacteria, with the *Prevotella*-dominated cluster containing the most genera, followed by the *Ruminococcus*- and *Paraprevotella*-dominated clusters. Both *Succiniclasticum*- and *Butyrivibrio*-dominated clusters had negatively correlated with other genera. In addition, 16 clusters were formed by two genera, including five clusters with a positive correlation between bacteria and bacteria, two clusters with a positive correlation between bacteria and fungi, two clusters with a negative correlation between bacteria and fungi, five clusters with a positive correlation between fungi and fungi, and two clusters with a negative correlation between fungi and fungi (Figure 4).

### Temporal dynamics of metabolic characteristics in the yak rumen microbiome

**Figure 5:**
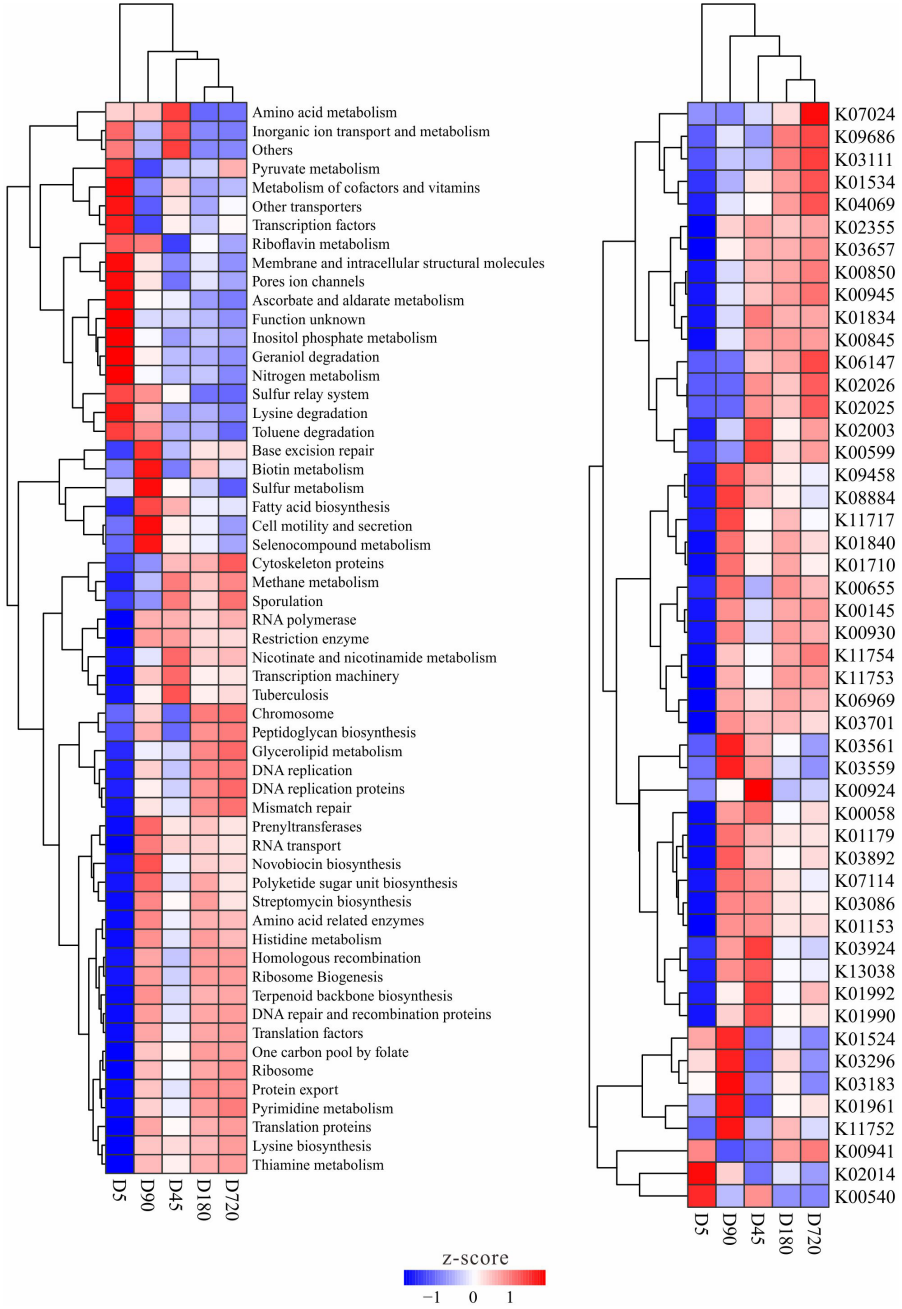
Metabolism and enzymes with significantly different abundance in the yak rumen at different ages.

The functions of the rumen microbiome in yaks were predicted by using PICRUSt, and functions with significantly different abundance among ages were detected. There was no metabolic that only significant enrichment in one or several groups, and some metabolites with significant differences in the abundance between different groups were also smaller in a multiplicity of differences. The abundance clustering results of differential metabolism and enzyme showed no difference between D180 and D720, and most of the differences existed with the D5, such as pyruvate, ascorbate, inositol phosphate, geraniol degradation nitrogen, lysine degradation, and toluene degradation metabolisms had a high abundance at the D5, while methane, nicotinate and nicotinamide, and glycerolipid, novobiocin biosynthesis, polyketide sugar unit biosynthesis, histidine and pyrimidine metabolisms were in high abundance in the other groups. Some metabolisms were also enriched at D45 and D90 groups, such as microbiome related to amino acid metabolism was abundant at the D45, and biotin, sulfur, fatty acid biosynthesis, and selenocompound metabolisms were more abundant at the D90. For enzymes, bacteria related to the iron complex outer-membrane receptor protein (K02014) were more abundant at the D5, and bacteria related to ubiquinone/menaquinone biosynthesis methyltransferase (K03183), acetyl-CoA carboxylase (K01961) and uracil reductase (K11752) were more abundant in the D90 group (Figure 5).

## Discussion

To evaluate the composition and temporal dynamics of rumen bacteria and fungi in Zhongdian yaks, the full length of microbiome of yaks at different ages were sequenced in this study. Our findings showed that yaks underwent a gradual process of change from birth to adulthood and that the bacterial and fungal community diversity in the rumen stabilized at 2 years of age. A rait-based method to explore the succession mechanism of the rumen microbial communities and their influence on host immunity to help better understand the functional roles of the microbiome.

Rumen microbial and fungal structure varies with the age, which may relate to diet digestion, altitude adaptability, and immunity strength of the yak. There were over 100 species shared between the rumen bacterial and fungal communities of young ruminants and the rumen microbiota of adult yaks (Figures 1A,B), suggesting that the rumen microbiota of calves were active and involved in the digestion of fibrous material. With the growth of yaks, the number of gut microorganisms with oxygen tolerance and flagella decreased gradually, while the number of microorganisms with slow growth and spore formation increased gradually (14). The diversity and richness of bacteria increased with age, and the horizontal component of the rumen fungal phylum in calves was the same as that in adult yaks, but the proportion was significantly different. This indicates that the rumen environment of grazing adult yaks, although more functional, is also a closed and independent ecosystem with more specific and homogeneous bacterial and fungal communities, compared to primary communities with more heterogeneity among the younger ages. The gradual increases in bacterial diversity in Holstein cattle and goats from birth to adulthood are associated with a gradual change in community diversity (8, 15). Interestingly, similar studies have been reported in recent years on the gut microbes of human infants. It has been found that the intestinal flora of infants starts from the early colonizers who are variable and good at rapid proliferation, but the functional traits gradually converge and stabilize in the first year of life; the gut microbiota adapts to the anoxic environment in the intestine and spreads among individuals through spores, while the taxonomic composition of the flora continues to change (16).

Dominant microbiome changed with the growth of yaks. At the D5, phylum bacterial Proteobacteria and fungal Basidiomycota were significantly higher than that in other ages, which was consistent with previous studies that facultative anaerobes *Proteobacteria* is more suitable for a fluid diet of earliest stage life (17). Xufeng et al. found the abundance of Basidiomycota increased with the proportion of dietary concentrate, suggesting that Basidiomycota are also suitable for a fluid diet (18). In addition, *Prevotella* was found in all age but varied in number and composition. With the decrease of Proteobacteria and Basidiomycota, the abundance of Prevotella and Thelebolus was increased at the D45, and *Prevotella* carries plenty of Carbohydrate-active enzyme (19), which may help Zhongdian yaks digest plant fiber and produce certain volatile fatty acids, enabling them to adapt to the high-altitude environment and high-fiber diet (20).

Fungal *Thelebolus* was the first reported in yak rumen, and its abundance reached a peak at the D90. The previous report showed that *Thelebolus* has the ability to produce anti-inflammatory exopolysaccharide (21), suggesting *Thelebolus* assists host immunity promotion. The abundance of *Luteolibacter* was also increased, which was also the first time reported in yak rumen. Marine bacterium *Luteolibacter algae* H18 can degrade fucoidan, which may enrich the feed degradation ability of Zhongdian yaks (22), while its positive partner ascochyta contains some disease-causing species (23). After 90 days, the diet was obtained entirely from free grazing and had more exposure to pathogens in the environment, like black leg causer *Plenodomus* was increased at 180 days age (24). *Plenodomus* is positively related to *Fretibacterium* and is also an opportunistic pathogen. There was no significant change in rumen bacterial structure between 180 and 270 days after birth, suggesting that the bacterial ecosystem tended to be stable after 180 days, while the fungal structure was changed as the proportion of *Penicillium* had increased significantly. *Penicillium* isolated from the gastric juice of cow rumen has a high capacity for cellulose degradation (25), assisting digest high altitude diet.

The strong functional correlation between rumen genes and microorganisms is evident in the synergistic effect observed during the early stages of rumen development (26). This study demonstrates an up-regulation of Transport and Catabolism in the 5-day-old and 180-day-old groups, indicating the importance of enhancing these processes for the maintenance of overall calf health. Newborn calves require strengthened transport and catabolism to ensure their well-being during this critical stage (27). Similarly, calves at 180 days of age face challenges such as cold winter conditions and a lack of forage, necessitating an increase in transport and catabolic activities. Concurrently, the Day5 group, representing early rumen development, exhibits a robust capacity for heterogenesis and metabolism. However, with advancing age, the rumen-based heterogenesis and related metabolism in Zhongdian yaks experience downregulation. Notably, the down-regulation of immune system-related genes observed may be associated with passive immunity acquired through colostrum intake (28).

With the change of diet, rumen microbial richness and diversity of calves changed to a mature ruminant state. The animals in this study were grazed in the wild for a long time without artificial feeding. Newborn calves began to eat the plant fiber slowly at an early stage to promote the development of the rumen and affect microbial colonization at the early stage of rumen development. The trend of diversity change was different between bacteria and fungi. The diversity index and number of bacterial OTUs communities increased with age, while the fungus reached the first peak at day 90, which was the time of weaning. The change in bacterial diversity is consistent with that in previous studies of Mongolian cattle data (29) and dairy calf data (13).

A limitation of this study might be related to the sample size. In the beginning, six underage yaks and six adult yaks were included in this study, but three underage yaks died during the experiment since a series of reasons. Although the difficulties of sampling collection of the wild animals were usually met, small sample size is a factor that might cause individual variation of our result. However, the basic dynamics of rumen microbiome from birth to adulthood are well investigated. More yaks and samples should be included in future studies to verify our results.

## Conclusion

Based on the full-length analysis of 16S rDNA and ITS sequences, we studied the dynamic changes of rumen microorganisms in grazing yaks at different growth stages. The rumen microbial community of yaks remained stable during growth and development, and we identified the signature rumen bacteria and fungi at each growth stage. Furthermore, we observed temporary changes in the characteristic bacteria and fungi in the two age compartments, which were linked to diet changes, rumen wall development, and microbial interaction. The colonization of rumen microflora in the early stage may influence the microbial community of yaks upon sexual maturity. Additionally, rumen fungi exhibited associations with bacteria at different growth stages. This study highlights the significance of rumen fungi and bacteria in grazing yaks.

## Data availability statement

The datasets presented in this study can be found in online repositories. The names of the repository/repositories and accession number(s) can be found in the article/Supplementary material.

## Ethics statement

The animal study was reviewed and approved by All animals used in this experiment were approved by the animal protection and utilization committee of Yunnan Agricultural University, China (protocol # 2017-0081), and there was compliance with the guidelines of the Laboratory Animal Ethics Committee in experimental animal handling. Written informed consent was obtained from the owners for the participation of their animals in this study.

## Author contributions

DW, HM, and SY made substantial contributions to the conception or design of the experiments. DW, GZ, JZ, and SY performed the experiments. DW and SH analyzed the data. JC and SY wrote the paper. All authors contributed to the article and approved the submitted version.

## Funding

This research was supported by the National Natural Science Foundation of China (32060762), Science Research Foundation of Education Department of Yunnan Province (2023J0518), Research Project of Department of Education of Guangdong Province (2022ZDZX4041), and Agricultural Basic Research Joint Project of Yunnan Province (202301BD070001-095).

## Conflict of interest

The authors declare that the research was conducted in the absence of any commercial or financial relationships that could be construed as a potential conflict of interest.

## Publisher’s note

All claims expressed in this article are solely those of the authors and do not necessarily represent those of their affiliated organizations, or those of the publisher, the editors and the reviewers. Any product that may be evaluated in this article, or claim that may be made by its manufacturer, is not guaranteed or endorsed by the publisher.
